# Prevalence and Etiological Factors of Dental Trauma among 12- and 15-Year-Old Schoolchildren of Lebanon: A National Study

**DOI:** 10.1155/2021/5587431

**Published:** 2021-03-05

**Authors:** Chirine Abdel Malak, Carole Chakar, Alain Romanos, Samar Rachidi

**Affiliations:** ^1^Clinical and Epidemiological Research Laboratory, Doctoral School of Science and Technology, AZM Center for Biotechnology, Tripoli, Lebanon; ^2^Department of Periodontology, Faculty of Dental Medicine, St Joseph University of Beirut, Beirut, Lebanon; ^3^Department of Periodontology and Implantology, Faculty of Dentistry, Lebanese University, Beirut, Lebanon; ^4^Clinical and Epidemiological Research Laboratory, Faculty of Pharmacy, Lebanese University, Hadath, Lebanon

## Abstract

**Background:**

Traumatic dental injuries represent nearly 5% of children and adolescents' injuries leading to serious medical and psychological issues. This current study aims to evaluate the prevalence of dental trauma and its potential association with different predisposing factors among 12-and 15-year-old schoolchildren in Lebanon.

**Materials and Methods:**

7902 schoolchildren, 3806 male and 4096 female aged 12 years (*n* = 3985) and 15 years (*n* = 3917), were recruited by a stratified multistaged randomized cluster sampling method from public and private schools and were clinically examined in a national cross-sectional study. WHO criteria were used to assess anterior permanent teeth; the nature of trauma, the tooth involved, the size of the incisal overjet, and the type of the lip coverage were furthermore assessed. Data regarding age, sex, and causes of TDI were recorded through a structured questionnaire.

**Results:**

The prevalence of dental trauma to anterior teeth was 10.9%. Maxillary central incisors (83.7%) were commonly affected. The most common type of injury was enamel fracture (68.3%), falls being the main reason (52.5%). Increased overjet (OR = 2.32, *p* = 0.034), deficient lip coverage (OR = 5.73, *p* = 0.019), and gender (OR = 5.36, *p* ≤ 0.001) were significant predisposing factors for dental trauma.

**Conclusion:**

This research highlighted many predisposing factors for dental trauma that affect commonly the anterior teeth. Based on these results, the implementation of strategic preventive measurements targeting especially the identified risk groups remains crucial.

## 1. Introduction

Traumatic dental injuries (TDIs) are listed among the most common injuries occurring during childhood and adolescence, highly vulnerable periods for dental trauma [1].

Approximately 25% of all schoolchildren and adolescents between 7 and 19 years have suffered from a type of TDI involving their permanent dentition [2, 3].

Nowadays, TDI is considered a challenging public health concern [4, 5] with a great impact on a person's quality of life since it could be a potentially life-changing accident.

According to epidemiological research, the prevalence of traumatic dental injuries varied from one country to another. Data showed that 6.1% to 41.6% of individuals encounter TDIs during childhood or adolescence [6]. This divergence may arise from the nature and approach of the study, the population size, the sampling method, the adapted classification and indices, and the cultural background that reflects the tendency of violence of the concerned country [6].

Major esthetic, functional, and therapeutic unpredictable riddles are associated with TDI leading to a multidisciplinary complex treatment plan [4]. Clinically, dental trauma can lead to crown and/or root fracture, tooth discoloration, pulp necrosis, apical periodontitis, root resorption, and fistulas.

Psychologically, children with TDIs commonly encounter emotional distress, influencing their self-esteem and quality of life [7–9]. Moreover, a relationship between TDI and academic achievement is proven [10].

Depending upon the severity, type, and duration of a TDI, the monetary cost of the required treatment can potentially be considerable [11]. In developing countries, where dental coverage is unavailable, this could be a major cause of oral health disparities.

The most commonly affected teeth with dental trauma are the maxillary central incisors, and the most frequent type of dental injury is enamel fracture followed by enamel and dentine fracture [12].

TDIs risk factors are not crystal clear; variation between countries, age groups, genders, and socioeconomic environments are well documented. Male gender, child age, greater overjet, inadequate lip coverage, anterior open bite, caries in the permanent dentition, overweight, maxillary incisor protrusion, a previous history of TDI, tongue piercing, the use of alcoholic beverages, and participation in sports are proved etiological factors of TDIs [13, 14]. Furthermore, the environmental component and individual behaviors account for a critical part of this multifactorial etiology [2].

Even though epidemiological studies on TDI have been conducted in many countries, Lebanon remains without any data. Given the adverse impact of TDI on the quality of someone's life, it is crucial to clarify its causes and risk factors, thus preventing its occurrence. In this perspective, the present study aims to evaluate the prevalence and epidemiological factors related to TDI among Lebanese schoolchildren.

## 2. Materials and Methods

This school-based national retrospective study was conducted in the six governorates of Lebanon, from September 2018 to June 2019, among 12-year-old (*n* = 3985) and 15-year-old (*n* = 3917) schoolchildren selected from public and private secondary schools located in the urban, suburban, and rural areas. It is a part of a Ph.D. research that assessed the oral health status and treatment needs of Lebanese adolescents. This study was approved by the ethics committee of the Azm Center for Research in Biotechnology and Its Applications, Lebanese University (document no. CE-EDST-1-2021). Informed consent was obtained from the participant's parents and schools.

Healthy, Lebanese schoolchildren, registered in grade VII or grade X in a public or private school with permanent dentition were the inclusion criteria. Non-Lebanese school-children, presence of deciduous teeth, permanent anterior teeth lost due to other causes than trauma (caries lesion), anodontia involving permanent anterior teeth, and children with general diseases were exclusion criteria.

### 2.1. Sample Size Calculation

The sample size was calculated based on the number of students in grade VII and grade X registered in private and public Lebanese schools according to the Ministry of Education and Higher Education. We estimated that a minimum sample size of 7752 children was required to achieve the level of precision with a standard error of 2% or less and the 99% confidence interval level and a prevalence of dental trauma of 50% used for the calculation. The decision to use a prevalence of 50% was due to the absence of data regarding TDI prevalence among Lebanese schoolchildren. However, any calculation using a different figure than 50% would require a smaller sample size to achieve the same precision [15].

Finally, 7902 school-children were included in the study.

### 2.2. Sampling Process

The sampling process was detailed in a previous publication [16]. Briefly, a stratified multistaged randomized cluster sampling method based on information provided by the Lebanese Ministry of Education and following WHO guidelines [17] was used. The six Lebanese governorates (Beirut, South of Lebanon, North of Lebanon, Nabatiyeh, Bekaa, and Mount Lebanon) were included. In the second stage, all high schools (clusters) in each governorate were listed; the number of schools selected by a simple random sampling was determined proportionally to the number of schools in the selected area. In the third stage, classes from the selected schools were chosen; the number of students that should be recruited in the private or public sector in each governorate was calculated proportionally to the total number of students in each sector. A random list was prepared for public and private schools separately. From these lists, the public and private schools were selected and asked for permission to recruit participants; examination proceeded from the first school to the next until getting the required sample from the location. It was determined that no more than 100 participants were to be recruited from a single school. The same procedure was followed in both public and private schools. In each case, when a school did not permit to recruit participants, the next school on the list was approached. The procedure used in the multicentric oral health survey [18] for the selection of classrooms within each school was also followed in the current study. If there was only one section for that age group, that class was included in the survey; if more than one section was in the required age group, a random selection method was used to further select the section to be examined. In some schools, the headteacher decided the classes and sections to be examined depending on the workload in their curriculum at the time of the survey.

### 2.3. Variables Measured

Data were recorded on a two-section standardized form by seven examiners. The first part gathered information on sociodemographic data (age, gender, school type, and location) although the second part was used to note oral examination data and contained information relevant to the injury accident (reason and location).

All participants received clinical examinations respecting the recommendations and methods of the WHO Oral Health Surveys [17]. Oral examinations were performed in the school's classrooms, in daylight upon using plane mouth mirrors. Involved tooth/teeth were noted.

TDI to anterior permanent teeth was recorded as recommended by the WHO as follow: 0 = no sign of injury, 1 = treated injury, 2 = enamel fracture only, 3 = enamel and dentine fracture, 4 = pulp involvement, 5 = missing tooth due to trauma, 6 = other damage, and 9 = excluded tooth [17].

Burden's criteria were used to assess lip coverage [19]: adequate lip coverage if the lip covered the upper incisors in the rest position, and inadequate lip coverage if a significant part of upper incisors was visible or lip strain was apparent during occlusion.

Following the WHO guidelines [17] and using the Community Periodontal Index (CPI) probe, maxillary overjet was measured with the teeth in centric occlusion; it is the distance from the labio-incisal edge of the most prominent maxillary incisor to the labial surface of the corresponding mandibular incisor. The overjet data were registered in two categories, ≤3 mm and >3 mm; the clinical examinations were performed by 7 examiners, and the intraexaminer variability was satisfactory (kappa = 0.82).

The cause of the injury was also noted. Fall, collision, traffic accident, violent incidents, unknown, and miscellaneous (biting on hard objects, inappropriate manipulation of teeth) were the given categories.

### 2.4. Statistical Analysis

Statistical Package for Social Sciences (SPSS) for Windows, version 20, was used to analyze the data. Frequencies and proportions of categorical variables were calculated. Multiple logistic regression analyses were used to assess the association between sociodemographic factors and clinical factors and dental trauma. Statistical significance was set at a value of less than 0.05.

## 3. Results

7902 participants (3806 boys and 4096 girls) were included in the study. The examined sample was almost equally distributed into individuals aged 12 years (50.4%) and 15 years (49.6%). 56.1% and 43.9% of the participants were recruited from private and public schools, respectively, and the vast majority live in a suburban area (63.0%). The prevalence of TDI was 10.4% with 823 children showing signs of previous TDI.

Table 1 summarizes the distribution of the TDI among the studied population and Table 2 shows the logistic regression analyses of the factors associated with dental trauma. The examination of TDI's pattern in relation to the age of the participants showed that TDI is more frequent in 15-year-old (11.1%) than in 12-year-old (9.8%) schoolchildren. Furthermore, gender analysis of the results reveals that the prevalence of TDI was higher in boys (17.5%) than girls (3.9%). Those results were statistically significant (*p* < 0.001). Boys were 5.36 times more likely to present dental trauma compared to girls (OR = 5.356, *p* < 0.001). 30% of children with inadequate lip coverage suffer from dental trauma, while only 7.9% of those with adequate lip coverage present TDI. Children with inadequate lip coverage were 5.73 times more likely to present dental trauma than children with adequate lip coverage. Regarding overjet (>3 mm vs. ≤3 mm), 29.5% and 8.04% exhibit TDI to the anterior teeth, respectively; these results are statistically significant (*p* = 0.034); schoolchildren with an overjet >3 mm were 2.319 times more likely to present dental trauma than children with an overjet ≤3 mm. Moreover, children from public schools were 1.29 times more likely to present dental trauma than children from private schools (OR = 1.29, *p* = 0.002).

Table 3 presents the causes of TDI in relation to gender. Data showed that, among both genders falling (53.6%) and sports (20.9%) were the most frequent etiologies behind traumatic dental injury; these results were statistically significant (Table 3). Collisions (13.4%), violent incidents (4.8%) and miscellaneous (1.8%) were less reported.

The majority of the accident that led to TDI took place at home (72%), followed by schools (21%) and then other locations as playgrounds and streets (7%).

In terms of genders, dental trauma was reported among 80.8% of boys and 19.1% of girls. The most frequent type of TDI was enamel fracture (68.0%), followed by enamel and dentin fracture (17.3%), and enamel and dentin with pulp involvement fractures (3.3%). 23 children (0.3%) have a missing tooth due to trauma and 90 (1.1%) have restorations highlighting a previous TDI treatment.

Table 4 shows the type of teeth involved in traumatic dental injuries according to gender. We recorded 1232 involved teeth among children with TDI. In a statistically significant manner (*p* = 0.018), boys (62.4%) more often had multiple teeth affected than girls (37.6%). The most commonly affected teeth were the maxillary central incisors (81.6%), followed by maxillary lateral incisors (12.7%) and mandibular central incisors (5.7%). The type of affected teeth was not statistically linked with any other variable.

## 4. Discussion

The present study was a national cross-sectional survey that aims to evaluate the prevalence and epidemiological factors associated with TDI among 12- and 15-year-old Lebanese schoolchildren. The adopted study design forced us to record only teeth-related injuries since soft tissue and bone injuries cannot be recorded at the time of the clinical examination because they do not exist anymore. Moreover, the study is based on self-reported information from adolescents who may mislead the investigator impulsively or consciously, especially when it comes to the cause of the TDI.

Based on the recommendation of the WHO, we targeted 12- and 15-year-old schoolchildren as during this period a large number of physiological and psychological events took place, leading children to be actively involved in a lot of physical activities. Furthermore, this allows the current results to be compared with previous international data. On an international scale, the prevalence of traumatic dental injuries varies significantly, ranging from 4% to 58% according to different epidemiological studies. In the current study, 10.4% of 12- and 15-year-old schoolchildren presented TDI. This result is comparable to different studies conducted in India [20], Brazil [21], Turkey [22], Canada [11], and Albania [23], on similar age groups. However, this proportion is higher than the 6.1% reported by Noori et al. in Irak [24], the 6.4% reported by Naidoo et al. in South Africa [25], and is interestingly lower than the 36% reported in Iran [26], 14.4% reported in India [27], the 37.9% reported in Chile [28], the 35.0% reported in Thailand [29] and the 39.5% reported in Saudi Arabia [30]. This wide divergence in the results could be attributed to many factors such as the study design, the inclusion/exclusion criteria, the sample size, the sampling procedure, the adopted diagnostic criteria, the chosen age groups, and the cultural and behavioral profile of each country.

In correlation with a recent systematic review and meta-analysis [6], the current study showed that the prevalence of TDI was higher among boys. The authors identified what appears to be a gender-based discrepancy in physical activity levels among youth; girls are less involved in physical activity than boys who tend to be more violent, energetic, and sportive. Researchers highlighted that females generally mature faster in certain cognitive and emotional areas than males during childhood and adolescence [31] leading to the vacation of widespread acts of direct and intense violence and promoting peaceful attitudes. Besides, the cultural and social aspect of the society have a vigorous impact on gender divergence in lifestyle and should not be underestimated.

The present research proved that in comparison with mandibular teeth, maxillary teeth are more involved in traumatic dental injuries with the maxillary central incisors being the most common traumatized teeth.

This is in correlation with existing literature [32] and may be related to the position of the maxillary incisors that could be at a higher risk if protruded or not covered by the lips. Also, injury to maxillary incisors is more common than mandibular incisors because the trauma to mandibular teeth is dissipated due to the flexible aspect of the mandible that is not rigidly attached to the cranial base [33]. In most cases, only one tooth was affected, which is also similar to findings reported in a previous study [34].

15-year-old children presented more TDI compared to 12-year-old children. Other previous studies showed comparable results [35, 36]. This can be explained by the fact that the exposure time to any type of injury is increased.

In the current study, a significant difference was found between the prevalence of TDI among children in private and public schools (9.1% and 12.1% respectively). Many studies discussed the relation between socioeconomic status and TDI and two controversial opinions exist. Many authors have reported that schoolchildren with lower socioeconomic thus attending public school status are more likely to suffer from TDIs [37]; other studies showed an inverse correlation, with children with higher socioeconomic status having a higher risk of TDIs [24]. This is elucidated by the fact that a higher socioeconomic level is correlated with greater access to physical leisure activities and equipment, such as bicycles, motors, and skateboards, which can expose to more accidents if used without safety precaution. Consequently, children's education about the importance of sound safety measures in sports venues accompanied by the use of protective athletic appliances such as mouth guards remain among suggestions that can be effective in reducing the incidence of dental trauma in this age range.

Schoolchildren with inappropriate lip coverage had a 5.73 times greater chance of having TDI. Similar results are showed in a recent systematic review and meta-analysis [14]. Lips play a crucial role in the protection of the underlying teeth; thus, inadequate lip coverage is considered a major risk factor for TDI.

Falls constituted the main etiological factor (53.6%), followed by sports (20.9%) and collisions (13.4%). Similar findings are reported in the literature [6, 38].

Violence was also among the causes of dental trauma in children and adolescents in the present study, it reflects the behavioral and emotional traits of this age group; in this regard, special attention should be given to the school and home social education, promoting a culture of self-control and social respect towards others. Moreover, we cannot pass across the possibility of domestic violence and abuse being an alarming and distressing etiological factor of TDI. In this perspective, a national public awareness campaign is of vital importance; they should be dedicated to getting these victims out of the shadows.

The majority of the injuries took place at home followed by school and other places such as playgrounds and streets. Similar results were obtained by other studies [39]. These findings are supported by the fact that nowadays schools are built to ensure safety and security to all children; those protective measures are not always present at home.

In the present investigation, enamel fracture (68%) was the most common TDI type. This finding corroborated those of previous studies [6]. The majority of affected subjects were present with untreated dental trauma and this is in line with many previous studies [40, 41]. The underestimation of oral health problems, in addition to the financial liabilities, may stand behind the high level of untreated patients.

In the present study, overjet more prominent than 3 mm was associated with a higher risk of TDI. This correlation was widely studied in different surveys without conclusive results; several studies have demonstrated this relationship [42, 43] while others did not find any correlation [44]. The interaction between oral risk factors and social and environmental behaviors may explain this divergence [2].

## 5. Conclusion

The current study is among the first national studies conducted in Lebanon that address the prevalence and epidemiological risk factors of TDIs. The elevated prevalence of traumatic dental injuries in addition to the high percentage of untreated children with TDIs stresses the need for increased awareness in the Lebanese population. Increased overjet, deficient lip coverage, and male gender were significantly associated with dental trauma. The implementation of educational programs oriented towards health policymakers, parents, and children is highly recommended. Screening school campaigns to identify groups with high anatomic and behavioral risk for TDI should be conducted, so relevant preventive measures such as preventive orthodontic treatment and use of mouth guards can be implemented.

## Figures and Tables

**Table 1:** Univariate analyses of the factors associated with presence of dental trauma.

	Children examined (%)	Presence of dental trauma
Age		
12 years	3984 (50.4%)	390 (9.8%)
15 years	3918 (49.6%)	433 (11.1%)
-*p* value		0.066

School		
Private	4434 (56.1%)	403 (9.1%)
Public	3468 (43.9%)	420 (12.1%)
-*p* value		<0.001

Gender		
Boys	3806 (48.2%)	665 (17.5%)
Girls	4096 (51.8%)	158 (3.9%)
-*p* value		<0.001

Location		
Rural	1811 (22.9%)	222 (12.3%)
Urban	1111 (14.1%)	109 (9.8%)
Suburban	4980 (63.0%)	492 (9.9%)
-*p* value		0.014

Lip coverage		
Adequate	7009 (88.6%)	555 (7.9%)
Inadequate	893 (11.4%)	268 (30.0%)
-*p* value		0.034

Overjet		
≤3 mm	7032 (89.0%)	566 (8.04%)
>3 mm	870 (11.0%)	257 (29.5%)
-*p* value		<0.001

**Table 2:** Logistic regression analyses of the factors associated with dental trauma.

Dependent variables	Independent variables	-*p* value	Unadjusted OR	95% CI for OR	
				Lower	Upper
Dental trauma	Aged 15 years	0.004	1.08	1.02	1.13
	Public school	0.002	1.29	1.10	1.53
	Location (ref: rural)	0.748			
	*Urban area*	0.640	0.94	0.72	1.23
	*Suburban area*	0.448	0.93	0.77	1.12
	Gender	≤0.001	5.36	4.47	6.42
	Lip coverage	0.019	5.73	4.36	6.18
	Overjet	0.034	2.32	1.26	3.56

**Table 3:** Causes of traumatic dental injuries according to gender.

Variables	Boys *n* (%)	Girls *n* (%)	Total *n* (%)
Falls	345 (52)	96 (60.8)	441 (53.6)^*∗*^
Collision	86 (13)	24 (15.2)	110 (13.4)
Traffic accident	23 (3.5)	6 (3.8)	29 (3.5)
Sports	146 (22)	26 (16.5)	172 (20.9)^*∗*^
Violent incidents	36 (5.5)	3(1.9)	39 (4.8)
Unknown	16 (2.0)	1 (0.6)	17 (2)
Miscellaneous	13 (2.0)	2 (1.2)	15 (1.8)
Total	665 (80.8)	158 (19.1)	823 (100)

^*∗*^ *p* < 0.05 statistically significant.

**Table 4:** Type of teeth involved in traumatic dental injuries according to gender.

Type of teeth	Boys *n* (%)	Girls *n* (%)	Total *n* (%)	*p*-value
Maxillary central incisors	638 (83)	368 (79.5)	1006 (81.6)	0.072
Maxillary lateral incisors	103 (13.3)	53 (11.4)	156 (12.7)	0.084
Mandibular central incisors	28 (3.7)	42 (9.1)	70 (5.7)	–
Mandibular lateral incisors	0 (0)	0 (0)	0 (0)	–
Maxillary and mandibular canines	0 (0)	0 (0)	0 (0)	–
Total	769 (62.4)	463 (37.6)	1232 (100)	0.018^*∗*^

^*∗*^ *p* < 0.05 Statistically significant.

## Data Availability

The datasets generated during and/or analyzed during the current study are available from the corresponding author on reasonable request.
